# A Comparative Study of Multiparametric MRI Sequences in Measuring Prostate Cancer Index Lesion Volume

**DOI:** 10.5334/jbsr.2832

**Published:** 2022-11-10

**Authors:** Omer Bagcilar, Deniz Alis, Mustafa Seker, Servet Erdemli, Umut Karaarslan, Aylin Kus, Cavit Kayhan, Yesim Saglican, Ali Kural, Ercan Karaarslan

**Affiliations:** 1 Silivri State Hospital, TR; 2 Acibadem Mehmet Ali Aydinlar University, School of Medicine, TR; 3 Acibadem Mehmet Ali Aydinlar University, TR

**Keywords:** Whole-mount pathology, prostate cancer, magnetic resonance imaging, comparative study

## Abstract

**Objectives:**

To compare the effectiveness of individual multiparametric prostate MRI (mpMRI) sequences—T2W, diffusion-weighted imaging (DWI) and apparent diffusion coefficient (ADC), and dynamic contrast-enhanced (DCE)—in assessing prostate cancer (PCa) index lesion volume using whole-mount pathology as the ground-truth; to assess the impact of an endorectal coil (ERC) on the measurements.

**Materials and Methods:**

We retrospectively enrolled 72 PCa patients who underwent 3T mpMRI with (n = 39) or without (n = 33) an ERC. A pathologist drew the index lesion borders on whole-mount pathology using planimetry (whole-mount_vol_). A radiologist drew the borders of the index lesion on each mpMRI sequence—T2W_vol_, DWI_vol_, ADC_vol_, and DCE_vol_. Additionally, we calculated the maximum index lesion volume for each patient (maxMRI_vol_). The correlation and differences between mpMRI and whole-mount pathology in measuring the index lesion volume and the impact of an ERC were investigated.

**Results:**

The median T2W_vol_, DWI_vol_, ADC_vol_, DCE_vol_, and maxMRI_vol_ were 0.68 cm^3^, 0.97 cm^3^, 0.98 cm^3^, 0.82 cm^3^, and 1.13 cm^3^. There were good positive correlations between whole-mount_vol_ and mpMRI sequences. However, all mpMRI-derived volumes underestimated the median whole-mount_vol_ volume of 1.97 cm^3^ (P ≤ 0.001), with T2W_vol_ having the largest volumetric underestimation while DWI_vol_ and ADC_vol_ having the smallest. The mean relative index lesion volume underestimations of maxMRI_vol_ were 39.16% ± 32.58% and 7.65% ± 51.91% with and without an ERC (P = 0.002).

**Conclusion:**

T2W_vol_, DWI_vol_, ADC_vol_, DCE_vol_, and maxMRI_vol_ substantially underestimate PCa index lesion volume compared with whole-mount pathology, with T2W_vol_ having the largest volume underestimation. Additionally, using an ERC exacerbates the volume underestimation.

## Introduction

Multiparametric magnetic resonance imaging (mpMRI) is the essential imaging tool in prostate cancer (PCa) diagnosis, with increasing prominence as recent guidelines suggest using mpMRI before systemic biopsy in men with a suspicion of PCa [1]. The primary treatment of PCa is radical prostatectomy, while eligible patients might opt for active surveillance or focal therapy [2], avoiding comorbidities related to radical prostatectomy [3]. The precise estimation of PCa index lesion volume on mpMRI is essential in guiding focal therapy since the overestimation of the lesion size increases the risk of complications, while the underestimation might lead to insufficient disease control.

To date, several studies have explored the accuracy of prostate mpMRI in measuring the PCa index lesion volume with contradictory results [2,4,5,6,7]. Some of the studies had flaws, including using the ellipsoid formula for determining the ground truth volume [4,8], using biopsy specimen volume as the ground truth [9], overlooking the individual performance of mpMRI sequences [10], and relying on the correlation without assessing actual volumetric differences [11,12]. Furthermore, there is little evidence for the impact of an endorectal coil (ERC) in estimating the index lesion volume [8].

In this work, we compared the effectiveness of prostate mpMRI sequences—T2-weighted imaging (T2W), diffusion-weighted imaging (DWI) and apparent diffusion coefficient (ADC) maps, and dynamic contrast-enhanced imaging (DCE)— in assessing the index lesion volume using whole-mount pathology as the ground-truth. Additionally, we evaluated whether using an ERC impacts the performance.

## Methods

The local ethics committee approved this study and waived the need for informed consent for the retrospective evaluation of anonymized medical data. We reviewed medical records of consecutive PCa patients who underwent prostatectomy in our institution between January 2019 and December 2020. The patients meeting the following inclusion criteria were identified: (1) diagnosis of PCa on whole-mount pathology; (2) having a digitized whole-mount specimen; (3) having a prostate mpMRI scan obtained at 3T mpMRI at our center three months before the radical prostatectomy. Initially, 87 patients were identified based on the inclusion criteria.

We excluded patients based on the following criteria: (1) patients with prominent post-biopsy hemorrhage obscuring the index lesion (n = 2); (3) patients with index lesion volume less than 0.5 mL on whole-mount pathology (n = 6). Furthermore, we excluded 3 PCa patients with a Gleason Score of 3 + 4 without any intraductal pattern or cribriform component as the index lesion was invisible on mpMRI.

### Prostate mpMRI

All patients underwent prostate mpMRI on a 3.0 Tesla MRI scanner (Skyra, Siemens Medical Systems, Germany); the mpMRI scans were performed with an 18-channel phased-array surface coil and a liquid perfluorocarbon-filled ERC (Medrad, Bayer). All mpMRI scans were acquired at least six weeks after the biopsy to minimize the negative influence of post-biopsy hemorrhage on the diagnostic evaluation. The prostate mpMRI protocol comprised tri-planar T2-weighted imaging, DWI, and DCE imaging from the first to the last. The detailed parameters regarding the MRI sequences are given in Supplementary Table S1.

### Index lesion volume on mpMRI

A radiologist with over 20 years of prostate imaging experience measured the volumes of the index lesion on mpMRI. To ensure that the lesions being measured were the same lesions on whole-mount pathology and mpMRI, we informed the radiologists regarding the location of the index lesion on whole-mount pathology using the sector map proposed by the PI-RADS [13]. The radiologist was blinded to the histopathological volume, yet we allowed the radiologist to evaluate all mpMRI sequences to simulate a clinical workflow following prior studies [5]. The radiologist was free to set the window level at their preference for each patient and sequence.

First, the radiologist manually drew the borders of the index lesion on T2W, DWI (b-value of 2000 s/mm^2^), ADC maps, and K^Trans^ maps on a dedicated workstation (Syngo Via, Siemens Medical Systems, Germany). Then, the software automatically calculated the index lesion volumes. These volumes were referred to as T2W_vol_, DWI_vol_, ADC_vol_, and DCE_vol_. Additionally, we determined the maximum index lesion volume from any of mpMRI sequences (i.e., T2W_vol_, DWI_vol_, ADC_vol_, and DCE_vol_) for each patient (maxMRI_vol_) [7]. For instance, for an individual patient, if the largest lesion size was measured on T2W_vol_, then that volume was selected in calculating maxMRI_vol_.

### Index lesion volume on Whole-mount pathology

A genitourinary pathologist evaluated all whole-mount specimens following the guidelines. The pathologist first assessed the macroscopic specimens visually, then 3 mm tissue blocks were created using a purpose-built mega cassette. The tissue blocks were embedded in paraffin, sliced at a thickness of 3 um per block (i.e., a single slice of 3 um was obtained per 3 mm block) perpendicular to the posterior plane, and stained with hematoxylin and eosin. Prepared micro-slices were mounted on a purpose-built mega slide, digitalized, and transferred to in-house software.

The genitourinary pathologist measured the index lesion using planimetry. The software automatically calculated the volume of the index lesion (whole-mount_vol_). We did not apply a correction factor for tissue shrinkage following prior work [7]. The lesion with the highest Gleason score was defined as the index lesion; if multiple lesions were present, the lesion with the largest volume was accepted as the index lesion [14].

### Statistical analysis

The statistical analyses were performed using the SciPy library of the Python programming language. The normally distributed continuous variables are presented using the mean and standard deviations. The continuous variables without a normal distribution are presented with the median and interquartile range (IQR), and the categorical and ordinal variables are presented with the frequencies and percentages. Next, the Spearman’s Rank-Order correlation was run to investigate the correlations between the histopathological and radiological volumes. Finally, we ran the Friedman and post-hoc Wilcoxon signed-rank tests to compare the index lesion volumes on whole-mount pathology and mpMRI.

The relative volumetric differences amongst mpMRI sequences using whole-mount pathology as the ground truth were assessed using the Friedman and post-hoc Wilcoxon signed-rank tests. Finally, the patients were divided respecting the use of an ERC, and the Man-Whitney-U test was run to compare the relative maxMRI_vol_ differences compared with whole-mount pathology. A P value < 0.05 was accepted as showing a significant result.

## Results

In all, 72 men with a mean age of 63.36 ± 11.25 years were enrolled in the study. The median whole-mount_vol_ was 1.97 cm^3^ (IQR, 3.35), while the median T2W_vol_, DWI_vol_, ADC_vol_, and DCE_vol_ were 0.68 cm^3^ (IQR, 1.15), 0.97 cm^3^ (IQR, 2.21), 0.98 cm^3^ (IQR, 2.21), 0.82 cm^3^ (IQR, 1.15) respectively. The Spearman test provided good positive correlations between whole-mount_vol_ and T2W_vol_, DWI_vol_, ADC_vol_, and DCE_vol_, with the r values of 0.75, 0.77, 0.78, and 0.75, respectively. The detailed characteristics of the study sample are given in Table 1.

**Table 1:** The characteristics of the study sample.


VARIABLES	FINDINGS

**Age *(years)***	63 ± 11.25

**PI-RADS score**	

PI-RADS 4	31 (43%)

PI-RADS 5	41 (57%)

**Lesion zone**	

Peripheral	60 (83%)

Transitional	12 (17%)

**Gleason score**	

3+4	44 (61%)

4+3	19 (26%)

4+4	1 (1%)

4+5	8 (11%)

**Prostate volume on MRI (*cm*^3^)**	37.26 ± 16.34

**Prostate volume on pathology (*ml*)**	45 ± 15

**PSA level (*ng/ml*)**	7.08 ± 5.02

**PSA density (*ng/ml*^2^)**	0.14 ± 0.12

**Endorectal coil**	39 (54%)


PI-RADS: Prostate Imaging Reporting & Data System; MRI: Magnetic Resonance Imaging; PSA: Prostate Specific Antigen.

**Figure 1:**
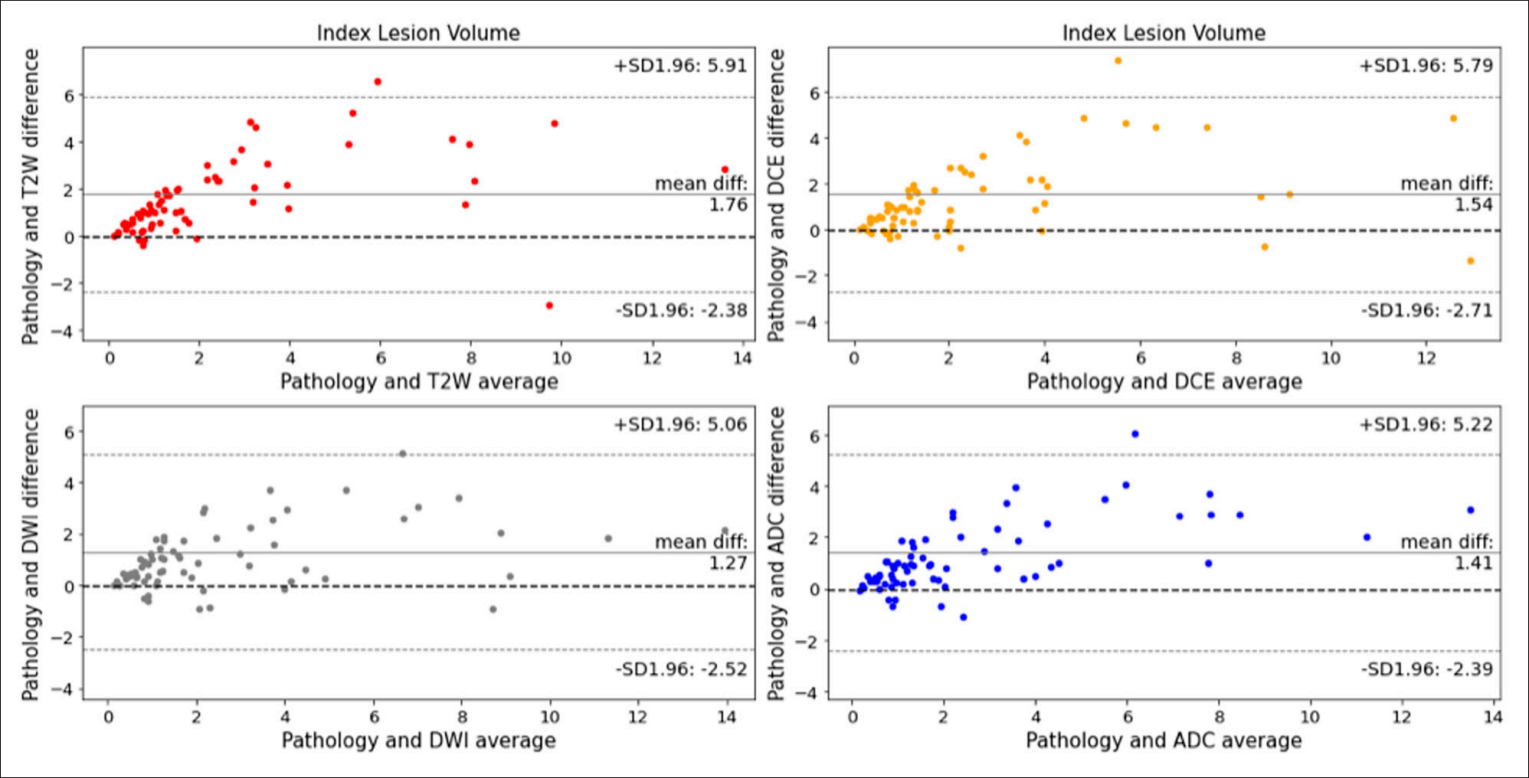
Bland-Altman plots comparing T2-weighted imaging (T2W_vol_), diffusion-weighted imaging (DWI_vol_), apparent diffusion coefficient (ADC_vol_), and dynamic contrast-enhanced (DCE_vol_) imaging in measuring prostate cancer index lesion volume compared with whole-mount pathology. All mpMRI sequences had a significant volumetric underestimation (P < 0.0001).

**Figure 2:**
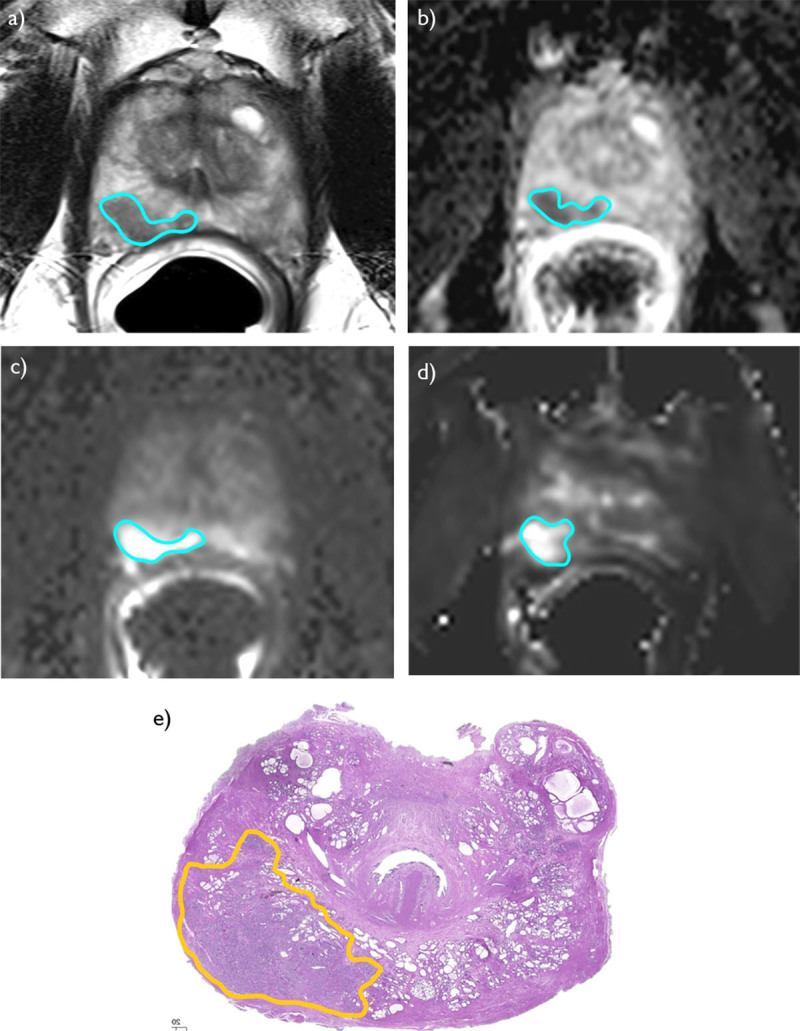
A 62-year-old man with Gleason Score 3 + 4 prostate cancer. The measurements of the index lesion volume on T2-weighted imaging **(a)**, apparent diffusion coefficient map **(b)**, diffusion-weighted imaging with a b-value of 2000 s/mm^2^ **(c)**, and the grayscale K^Trans^ map of dynamic contrast-enhanced imaging **(d)** are shown. In addition, the reference digitized whole-mount pathology slice is depicted **(e)**.

The Friedman test demonstrated that mpMRI-derived volumes were significantly lower than the whole-mount_vol_ (P = 0.001) (Table 2). The intra-patient differences of T2W_vol_, DWI_vol_, ADC_vol_, and DCE_vol_ with respect to whole-mount_vol_ are shown with the Bland-Altman plots in Figure 1. Figure 2 depicts index lesion measurements on mpMRI and digitized whole-mount pathology in a PCa patient.

**Table 2:** The index lesion volumes on whole-mount pathology and multi-parametric MRI.


VARIABLES	ABSOLUTE INDEX LESION VOLUME (CM^3^)*	RELATIVE VOLUME DIFFERENCE (%) **	P VALUE***

Whole-mount pathology	1.97 (3.35)	NA	NA

T2-weighted imaging	0.68 (1.15)	54.32% ± 32.84%(–67.27% – +98.48%)	<0.0001

Diffusion-weighted imaging	0.97 (2.21)	35.30% ± 41.21%(–105.08% – 95.96%)	<0.0001

Apparent diffusion coefficient map	0.98 (2.21)	35.96% ± 40.42%(–121.82% – +95.25%)	<0.0001

Dynamic contrast-enhanced imaging	0.82 (1.15)	41.98 ± 39.83%(–72.73% – 95.76%)	<0.0001

Maximum volume on MRI	1.13 (2.35)	24.72 ± 45.09%(–121.82% – 95.25%)	<0.0001


* Presented with a median and interquartile range; ** Presented with a mean, standard deviation, and range; *** Reflect the comparisons between the index lesion volumes on whole-mount pathology and respective mpMRI sequence.

The mean relative index lesion volume underestimation for T2W_vol_, DWI_vol_, ADC_vol_, and DCE_vol_ compared with whole-mount_vol_ were 54.32% ± 32.83% (range, –67.27 – +98.48), 35.3% ± 41.21% (range, –105.08 – +95.06), 35.96% ± 40.41% (range, –121.82% – +95.25%), 41.98% ± 39.82% (range, –72.73 – +95.76).

The Friedman test showed a significant difference in the relative index lesion volume underestimation amongst sequences (P = 0.001). DWI_vol_ and ADC_vol_ had a lower mean relative index lesion volume underestimation than T2W_vol_ (P = 0.001) and DCE_vol_ (P = 0.001 and 0.005). Additionally, DCE_vol_ demonstrated a lower relative volume underestimation than the T2W_vol_ (P = 0.001).

The mean relative index lesion volume underestimations of maxMRI_vol_ were 39.16% ± 32.58% (range, –63.38% – +81.53%) and 7.65% ± 51.91% (range, –121.81% – +95.25%) with and without an ERC, respectively (P = 0.002).

## Discussion

The main findings of the study are as follows: (1) T2W_vol_, DWI_vol_, ADC_vol_, and DCE_vol_ show a good correlation with whole-mount pathology in measuring the index lesion volume; (2) all mpMRI sequences underestimate the index lesion volume compared with whole-mount pathology; (3) T2W_vol_ leads to the largest index lesion volume underestimation, while DWI_vol_ and ADC_vol_ have the smallest; (4) notably, mpMRI with an ERC leads to a more significant index lesion volume underestimation.

The findings of the present work contrast with the initial studies on the performance of prostate mpMRI in measuring the index lesion volume, which documented that mpMRI overestimated the index lesion volume [11,15]. Nevertheless, these studies were carried out before the PI-RADS era and had flaws, as mentioned in the introduction. Further, prostate MRI have improved dramatically over the last decade. Indeed, recent studies document that mpMRI underestimates the index lesion volume [16,17]. Nevertheless, relatively few studies explicitly investigated which mpMRI sequence is the most effective in assessing PCa index lesion volume.

Cornud et al. [17] investigated the performance of the individual mpMRI sequences in assessing PCa index lesion volume in patients who underwent prostate mpMRI at 1.5T with an ERC. Similar to our results, the authors observed that ADC had the best performance in estimating PCa index lesion volume. DCE provided a worse performance than T2-weighted imaging. The authors argue that the poor performance of DCE might relate to the artifacts due to post-biopsy hemorrhage. In addition, we argue that field strength differences between studies might also contribute to these discrepancies.

Bratan et al. [7] and Sun et al. [18] showed that all mpMRI sequences underestimated PCa volumes, with maxMRI_vol_ providing the most accurate estimations. However, Bratan et al. and Sun et al. showed that DCE provided a better index lesion volume estimation than T2-weighted imaging and ADC. Unlike the present work, Sun et al. [18] measured the maximum enhancement area instead of kinetic parametric maps, probably leading to differences between the studies.

Le Nobin et al. [8] also showed that mpMRI significantly underestimated PCa volumes, with T2-weighted imaging providing a better volume estimate than ADC, yet contrasting with our findings. The authors argued that including patients with Gleason Score 3 + 3 PCa in their sample might be the underlying factor. Furthermore, they measured PCa foci other than the index lesion, which we assume might explain the inconsistency.

A few investigators compared the performance of mpMRI and prostate-specific membrane antigen positron emission tomography-computed tomography (PSMA-PET/CT) in estimating PCa volume [19,21]. For instance, Bettermann et al. [19] found that mpMRI significantly underestimated the index lesion volume, whereas PSMA-PET/CT provided a better estimation compared with whole-mount pathology. Likewise, Zamboglou et al. [20] also found that mpMRI underestimated the index lesion volume on whole-mount pathology. The authors demonstrated that the fusion of mpMRI and PSMA-PET/CT provides a better estimate compared with both modalities alone.

It is recognized that an ERC might deform the prostate gland, which might lead to differences in index lesion volume. Nevertheless, few earlier studies explicitly investigated this disadvantage. In accordance with our results, Bratan et al. [7] found that mpMRI at 3T with an ERC led to reduced performance in assessing PCa volume. Though it might be expected due to potential gland deformation of an ERC, the low performance of mpMRI with an ERC compared without an ERC in assessing PCa index lesion volume was a noteworthy finding.

Several limitations to the present work should be acknowledged. First and foremost, this was a single-center study with all mpMRI scans obtained from a single 3T MRI scanner, which hinders the generalizability. Second, we had a relatively low sample size, preventing us from investigating whether mpMRI-derived index lesion volumes vary across PI-RADS or Gleason scores or lesion location.

Third, we did not apply any correction factor for tissue shrinkage in the present work. Prior studies implemented a broad range of correction factors changing from 1 to 1.5 for compensating tissue shrinkage on whole-mount pathology [10,11,21,22,23]. On the other hand, in line with our work, some other authors argued that it was not possible to find an optimal correction factor for tissue shrinkage as it varies from patient to patient [7,17]. Nevertheless, mpMRI-based volumes underestimated the index lesion volume on whole-mount pathology without any correction factor in the present work. Thus, applying a shrinkage factor would only have exacerbated the volume underestimation. Hence, we suggest that this limitation does not hamper the findings of the present work.

In conclusion, the findings of the present work highlight that mpMRI-derived volumes, T2W_vol_, DWI_vol_, ADC_vol_, DCE_vol_, and maxMRI_vol_ underestimate PCa index lesion volume compared with whole-mount pathology, with T2W_vol_ having the largest volume underestimation and maxMRI_vol_ having the smallest. Therefore, using maxMRI_vol_ appears reasonable in assessing PCa index lesion volume. Additionally, using an ERC exacerbates the volume underestimation, and extra care should be exercised while estimating PCa index lesion volume on mpMRI with an ERC.

## Additional File

The additional file for this article can be found as follows:

10.5334/jbsr.2832.s1 Table S1. The detailed prostate multi-parametric magnetic resonance imaging parameters.
