# Supplementary material for: A Comparative Study of Multiparametric MRI Sequences in Measuring Prostate Cancer Index Lesion Volume
Source: J Belg Soc Radiol. 2022 Nov 10;106(1):105. doi: 10.5334/jbsr.2832 (PMC9650977; doi:10.5334/jbsr.2832)
Supplement: Table S1. — The detailed prostate multi-parametric magnetic resonance imaging parameters. [file jbsr-106-1-2832-s1.pdf]

Table S1. The detailed prostate multi-parametric magnetic resonance imaging parameters.

**Sequences**	**TSE T2-weighted imaging**			**Diffusion-weighted imaging**	**T1-weighted dynamic contrast-enhanced imaging**
Plane	Axial	Sagittal	Coronal	Axial	Axial
TR (msec)	5500	5040	3900	4800	4.81
TE (msec)	104	115	117	63	1.74
Time of acquisition (minutes)	3.21	2.33	3.05	4.02	2.52
FOV (mm)	200	220	240	200	260
Slice thickness (mm)	3	3	3	3	3
b-values (s/mm^2)	-	-	-	0, 50, 500, 1000	-
Matrix size	384x307	384x288	448x291	114x88	192x138

TSE: Turbo spin-echo; TR: Repetition time; TE: Echo time; FOV: Field of view.
